# A Reinforcement Learning Approach to Robust Scheduling of Permutation Flow Shop

**DOI:** 10.3390/biomimetics8060478

**Published:** 2023-10-07

**Authors:** Tao Zhou, Liang Luo, Shengchen Ji, Yuanxin He

**Affiliations:** Ministry of Education Key Laboratory of High Performance Ship Technology, Wuhan University of Technology, Wuhan 420100, China; a15630455659@163.com (T.Z.); jsc@whut.edu.cn (S.J.); 305364@whut.edu.cn (Y.H.)

**Keywords:** permutation flow shop, scheduling, deep reinforcement learning, disjunctive graph, policy network

## Abstract

The permutation flow shop scheduling problem (PFSP) stands as a classic conundrum within the realm of combinatorial optimization, serving as a prevalent organizational structure in authentic production settings. Given that conventional scheduling approaches fall short of effectively addressing the intricate and ever-shifting production landscape of PFSP, this study proposes an end-to-end deep reinforcement learning methodology with the objective of minimizing the maximum completion time. To tackle PFSP, we initially model it as a Markov decision process, delineating pertinent states, actions, and reward functions. A notably innovative facet of our approach involves leveraging disjunctive graphs to represent PFSP state information. To glean the intrinsic topological data embedded within the disjunctive graph’s underpinning, we architect a policy network based on a graph isomorphism network, subsequently trained through proximal policy optimization. Our devised methodology is compared with six baseline methods on randomly generated instances and the Taillard benchmark, respectively. The experimental results unequivocally underscore the superiority of our proposed approach in terms of makespan and computation time. Notably, the makespan can save up to 183.2 h in randomly generated instances and 188.4 h in the Taillard benchmark. The calculation time can be reduced by up to 18.70 s for randomly generated instances and up to 18.16 s for the Taillard benchmark.

## 1. Introduction

The Job-Shop Scheduling Problem (JSSP) stands as a renowned combinatorial optimization challenge within the realms of computer science and operations research, finding widespread application across industries such as manufacturing and transportation [1,2]. Workshop scheduling, through judicious allocation of pending tasks within a designated timeframe, facilitates optimal resource utilization, thereby aiding enterprises in mitigating excessive investments in raw materials, energy, and productivity. Moreover, the application of various algorithms has led to a reduction in the practical application costs associated with workshop scheduling, garnering substantial attention from scholars [3], engineering professionals [4], and manufacturers [5]. Notably, permutation flow shop workshops, emblematic of a prototypical workshop configuration, find extensive prevalence in manufacturing and large-scale product fabrication. [6,7,8]. In PFSP, there are n jobs ${\;J}_{1},\;\ldots ,{\;J}_{n}$, each of which consists of a sequence of m processes. There are m machines $M_{1},\;\ldots ,\;M_{m}$, $Q_{ij}$ is the ith process of Job ${\;J}_{i}$, and each process $Q_{ij}$ can only be performed by $M_{j}$. Moreover, the execution of any process cannot be interrupted nor preempted, and job delivery is not allowed. That is, the jobs must be executed in the same order on each machine. Furthermore, the PFSP has been established as an NP-hard conundrum [9], implying its intractability in yielding optimal solutions within polynomial time. Hence, the pursuit of judicious algorithmic design, generating high-quality solutions within acceptable timeframes for practical scenarios, assumes significant research import. Presently, the dominant methodologies for addressing this domain encompass exact algorithms [10], heuristic algorithms [11], metaheuristic algorithms [12,13], and deep reinforcement learning (DRL) algorithms [14]. Nevertheless, the existing mainstream approaches fall short of striking an optimal balance between solution quality and computational time. In light of this, we proffer an innovative end-to-end model based on DRL to effectively tackle this intricate predicament.

The PFSP, renowned as a challenging NP-hard endeavor, has yielded prolific research outcomes [15,16,17]. However, as production scales expand, exact algorithms such as integer programming models and branch-and-bound techniques struggle to provide timely resolutions for large-scale manufacturing quandaries. Over the past decades of inquiry, endeavors have been directed towards expeditiously deriving scheduling solutions through heuristic approaches. In the realm of heuristic methodologies targeting the optimization of maximum completion time for addressing PFSP, NEH has emerged as a paragon of efficiency [18,19], commanding the admiration of heuristic-minded scholars. In this context, Christos Koulamas [20] introduced a facile constructive heuristic algorithm aimed at the objective of maximum completion time, adeptly generating non-permutative schedules where advantageous, and demonstrating superior performance to the NEH algorithm in addressing the flow shop scheduling challenge. Zheng and Wang [21], in a fusion of the NEH heuristic with genetic algorithms, advanced an effective hybrid heuristic for the flow shop scheduling issue, substantiating its efficacy through empirical validation. Nagano et al. [22] introduced an N&M algorithm that penalizes the priority of NEH jobs based on the lower bound of job waiting times, thus reshuffling the initial sequence. Empirical findings underscore that the N&M algorithm secures superior outcomes to the NEH algorithm without escalating computational complexity. For the minimization of total tardiness in the context of flow shop scheduling, Fernandez-Viagas and Framinan [23] harnessed an NEH-based heuristic to unravel the quandary. Delving into decision problems contingent on job due dates, they delineated parallels with various associated decision challenges. Kalczynski et al. [24] propounded novel priority sequencing and coupled it with an uncomplicated disruption rule, resulting in a methodology that outperforms the NEH algorithm across all problem scales. In a bid to minimize total flow time in DPFSP, Pan et al. [25] extended the utility of NEH and LR through the introduction of three heuristic approaches, DNEH, DLR, and DLR-DNEH, effectively broadening the scope of the NEH application to alternative problem formulations and objectives.

Traditional heuristic methods are confined to addressing smaller-scale flow shop scheduling quandaries. Subsequently, to enhance computational efficiency and refine outcomes, numerous scholars have harnessed metaheuristic algorithms for tackling a diverse array of large-scale scheduling challenges. Leveraging their robust global search capabilities and relatively acceptable solution speeds, metaheuristic algorithms find application across both static and dynamic problem domains [26,27,28], emerging as the most prolific category in contemporary workshop scheduling research. In 2013, Ceberio et al. [29] proposed a hybrid approach consisting of a new estimation of the distribution algorithm and a variable neighborhood search. Conducted experiments demonstrate that the proposed hybrid approach obtains new best-known results in 152 cases. In 2015, Sayoti and Essaid Ri [30] introduced the Gold Ball Algorithm, a metaheuristic approach founded on football-inspired concepts for resolving flow shop scheduling problems. In 2016, Santucci et al. [31] proposed a new discrete Differential Evolution algorithm for the Permutation Flowshop Scheduling Problem with the total flowtime and makespan criteria. The core of the algorithm is a distance-based differential mutation operator defined by means of a new randomized bubble sort algorithm. In 2017, Dubois-Lacoste et al. [32] proposed the utilization of local search techniques to enhance the partial solutions derived from iterated greedy algorithms, applying this framework to the PFSP while aiming to minimize the maximum completion time. Empirical findings substantiated the advantageous nature of reoptimizing partial solutions. In 2018, Baioletti et al. [33] introduced a decomposition-based algebraic evolutionary algorithm for multi-objective permutation-based problems (MOEA/DEP). In order to mitigate the diversity loss during the evolution, MOEA/DEP introduces some additional components and variants. In 2020, Kaya et al. [34] formulated and compared five distinct methods for generating initial populations, employing a hybrid firefly-particle swarm optimization algorithm to evaluate the effects of various initial populations in tackling intricate flow shop scheduling dilemmas. In 2022, Li et al. [35] introduced an improved simulated annealing algorithm grounded in solution space pruning to address large-scale PFSP, concurrently presenting a hybrid release strategy based on the Palmer algorithm.

Given the fixed structure of heuristic and metaheuristic algorithms, the search performance encounters certain limitations. Many researchers have endeavored to harness machine learning algorithms for solving scheduling predicaments, owing to their potent learning capabilities that autonomously seek optimal solutions. DRL, a subset of machine learning, showcases robustness by eschewing the need for prior knowledge or fixed models. It attains experiential knowledge through interactions with the environment, thereby autonomously acquiring adept solutions, rendering the pursuit of optimal solutions more intellectually adept. Scholars, commencing in 2018, have embarked on infusing DRL into the arena of workshop scheduling. Pan et al. [36] employed a DRL paradigm rooted in policy gradients (PG), utilizing classical pointer networks as actors and multi-head attention networks as critics, underpinned by immediate rewards corresponding to completion times. Ingimundardottir et al. [37] introduced an imitation learning algorithm to acquire scheduling rules. However, due to the elevated time complexity inherent in workshop scheduling dilemmas, obtaining a multitude of optimal solutions for training purposes on large-scale problems proves impractical, thus constraining the method’s applicability. Lin [38] proposed a Deep Q-Networks (DQN)-based algorithm for addressing workshop scheduling, with the action space represented as a set of priority dispatching rules. At each state, the agent selects a rule. Yang et al. [39] established a mathematical model for dynamic PFSP utilizing DRL, extracting five distinct features as the state space and deploying the A2C algorithm to train a network in selecting appropriate heuristic rules. This, in essence, achieves a metaheuristic approach. Han and Yang [40] delved into the extraction of processing state information using convolutional neural networks and employed D3QN to train Q-values for diverse heuristic rules across distinct states. Despite the substantial achievements attained through the application of DRL to workshop scheduling, research specifically addressing PFSP using DRL methodologies remains comparatively limited.

Following substantial interactions with the environment, DRL models achieve the capability for iterative decision-making, accompanied by commendable generalization prowess. In comparison with heuristic and metaheuristic algorithms, the beauty of DRL lies in its capacity to resolve problems of all magnitudes through a single training iteration, obviating the need for recurrent training necessitated by varying problem scales [41]. Presently, DRL algorithms have progressively ascended as the mainstream approach to tackling combinatorial optimization quandaries. Indicative of this shift, certain investigations applying DRL to combinatorial optimization underscore the surpassing of metaheuristic algorithmic outcomes in certain domains [42]. However, existing endeavors employing DRL for resolving the PFSP remain beset by certain concerns. Foremost, it is very important to define the permutation flow shop scheduling system as the state in the Markov decision process (MDP). Nonetheless, prevalent approaches predominantly adopt mathematical models for state representation, inadvertently omitting a comprehensive and rational encapsulation of the scheduling environment’s entirety. Furthermore, the efficacy of information extraction from current states directly influences the training process of learning algorithms. Regrettably, the conventional feedforward neural networks, extensively favored in extant research, prove inadequate in efficiently extracting state information. In addition, existing research largely relies on DQN to train policy networks [43]. However, it is noteworthy that DQN does not inherently optimize policies, thereby potentially introducing instability or protracted convergence periods into the training dynamics.

To address the aforementioned quandaries, we present an end-to-end DRL approach to solve the PFSP. In pursuit of a more comprehensive portrayal of PFSP scheduling states, we ingeniously employ disjunctive graphs to represent the intricate tapestry of the PFSP scheduling landscape. To harness the wealth of information implicit in the underlying topological structure of these disjunctive graphs, we craft a policy network based on graph isomorphism network (GIN) for embedding and training it using the proximal policy optimization (PPO). In this construct, the policy network initially leverages a graph encoder to embed the multifaceted information contained in the disjunctive graph. Thereafter, an action selection network furnishes the agent with the optimal action through a probability distribution over available actions. We subjected our methodology to rigorous comparison with six baseline methods. Empirical findings consistently underscore the superior performance of our model in terms of both completion time and computational efficiency. Furthermore, even when confronting larger-scale instances, our model elegantly demonstrates robust generalization capabilities. This endeavor yields a manifold contribution, encapsulated as follows:

(1). A MDP model has been established for PFSP, elaborating in detail the construction of state space, action space, and reward scheme. Furthermore, an innovative application of disjunctive graphs encapsulates the state intricacies inherent in the scheduling domain.
(2). To more effectively extract information embedded within the graphical state structures, a policy network grounded in GIN has been introduced. Internally, this policy network employs a graph encoder to articulate the state representation, subsequently guiding decision-making based on the encoded state. The efficacy of this network has been validated through the resolution of diverse-scale instances.
(3). A novel end-to-end DRL paradigm has been advanced to address PFSP, surmounting the historical limitations in terms of generalization capacity. This model transcends prior constraints, enabling the resolution of problems of arbitrary dimensions after a single training iteration.

The remaining sections of the study are presented as follows: Section 2 provides a mathematical exposition of the PFSP and an introduction to the techniques employed. In Section 3, our research methodology is expounded, encompassing the establishment of the MDP model and the formulation of the policy network. Section 4 delineates the experimental protocol and engages in a comprehensive discourse on the findings. Lastly, Section 5 furnishes our conclusions, highlights the study’s limitations, and illuminates avenues for future exploration.

## 2. Problem Background

### 2.1. The Description of PFSP

This study delves into the PFSP, wherein a set of *n* jobs $J=\{J_{1},\;J_{2},\;\ldots ,{\;J}_{n}\}$ undergo processing across *m* machines $M=\{M_{1},\;M_{2},\;\ldots ,{\;M}_{m}\}$ through a sequence of processes $\left\{O_{i1},\;O_{i2},\;\ldots ,{\;O}_{im}\right\}$. The essence of this study revolves around orchestrating an optimal arrangement where all jobs are processed on each machine in a uniform sequence. Assuming that the jobs are processed in the order of machines 1 to *m*, let the job processing sequence be denoted by $\pi =\{\pi_{1},\;\pi_{2},\;\ldots ,\;\;\pi_{n}\}$. Our focus, in this discourse, centers on the minimization of the maximal processing time, serving as the bedrock of our scheduling objective. Within this context, the ensuing assumptions are set forth:

(1). A job can be processed on only one machine at any given moment;
(2). Jobs are independent and arrive at time zero without any disturbances during production;
(3). Once a job is initiated on a machine, it proceeds without interruption;
(4). Setup and transportation times between processes are encompassed within the processing duration;
(5). Each job is processed exactly once on each machine;
(6). The processing durations for all jobs on all machines are known in advance.

### 2.2. Disjunctive Graph

**Figure 1:**
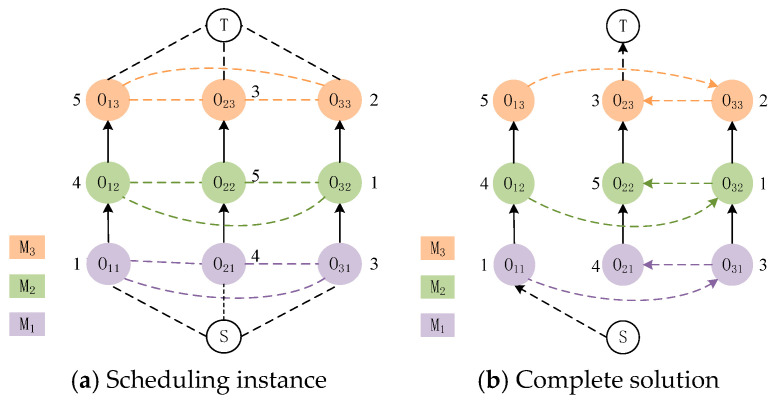
Disjunctive graph representation of 3 × 3 scheduling instance and its solution.

The disjunctive graph [44] consists of three components: vertices, connecting arcs, and disjunctive arcs. In order to provide a more comprehensive and coherent representation of the scheduling state for the permutation flow shop, we introduce the disjunctive graph to depict the scheduling process of the permutation flow shop. The disjunctive graph G=(O, C, D) stores data in the form of a graph structure, where O represents the set of nodes, with each node denoting a production process. C is a set of directed edges represented by solid lines, referred to as connecting arcs. The direction of each edge signifies the sequential constraint between processes of the same job. D represents a set of undirected edges depicted by dashed lines, known as disjunctive arcs, connecting nodes that need to be processed on the same machine. Once we establish the direction of each disjunctive arc, which indicates the processing order on each machine, we obtain a solution. *S* and *T*, respectively, denote the start and end of the schedule. Figure 1 illustrates an example of representing the scheduling state of the permutation flow shop using a disjunctive graph. In Figure 1a, we give the disjunctive graph representation for a workshop scheduling problem with three machines and three jobs. In Figure 1b, we depict a feasible solution for the disjunctive graph representation of the problem. Notably, in Figure 1a, undirected dashed lines connect processes of different jobs, while edges of different colors represent distinct machines. Among them, each column of the disjunctive graph represents different machining processes between the same job, and each row represents the same machine. In Figure 1b, a solution has been determined, resulting in directed edges throughout the graph.

**Figure 2:**
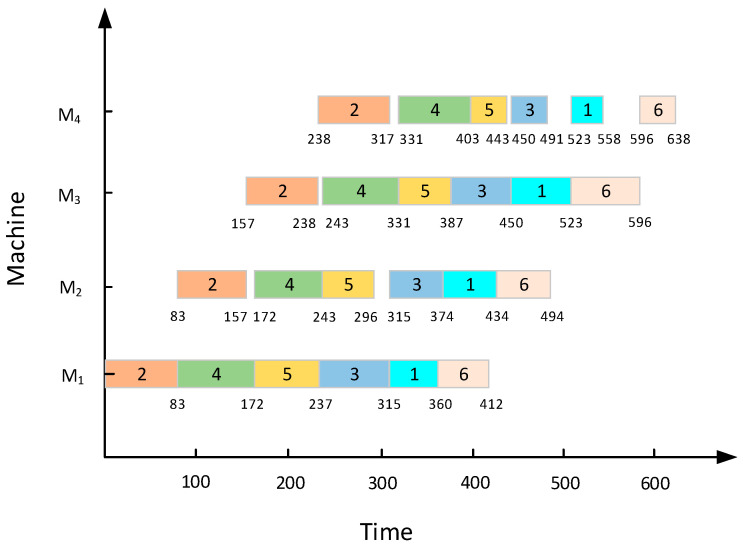
The scheduling instance of 6 × 4.

To elucidate the scheduling procedure of PFSP more lucidly, a permutation flow shop with six jobs and five machines is shown in Figure 2, for example, as can be seen from the Gantt chart, when the input jobs sequence π=(2,4,5,3,1,6), the makespan of this instance is the smallest, which is 638.

## 3. Methods

In this section, we shall elucidate the fundamental principles of our approach. First, we establish an MDP model based on the PFSP, elaborating in detail the methods for defining states, actions, state transitions, rewards, and policy. Subsequently, we devise an innovative strategy representation technique founded upon graph neural networks, encompassing the construction of both a graph encoder and an action selection network. Last, we present the training framework for our algorithm and provide a detailed exposition of the specific training regimen.

### 3.1. MDP Model

State: the PFSP is characterized by uniform processing procedures for all jobs, ensuring a consistent order of job processing on each machine. Upon selecting a job, the system can ascertain its completion time on each machine. We define a state as the disjunctive graph representing the scheduling system at each moment. Specifically, the initial state $s_{0}$ of each solution, iteration is denoted as in Figure 1a from Section Two. This graph encompasses both directed and undirected edges, with distinct-color nodes in each column representing process steps for the same job. Directed edges between nodes denote sequential constraints between process steps, while dashed lines connecting nodes in the same row signify undirected edges. These undirected edges link process nodes of the same color, signifying the need for processing on a shared machine. As the agent makes sequential selections from the candidate set of jobs and as the processing of certain steps of the preceding job concludes, the direction of the disjunctive arc between the two job nodes can be determined. Through successive decisions by the agent, the disjunctive graph progressively evolves from a mixed graph into a directed acyclic graph, illustrated in Figure 1b. This transformation signifies that with changing scheduling dynamics at each decision step, the disjunctive graph can offer distinct state compositions for the scheduling environment of PFSP. In turn, shifts in the scheduling environment will consequently yield varying disjunctive graphs.

Action: the effectiveness of action design directly influences the algorithm’s efficiency. Each action yields optimization benefits within distinct production environments, necessitating multifaceted considerations to minimize idle time between machines and enhance machine utilization. $a_{t}$ is the action taken by the agent in step t to select which job to enter the permutation flow shop. Due to priority constraints, only one job can be scheduled at a given moment. Hence, the agent’s action $a_{t}$ at step t corresponds to the remaining number of jobs in the state $s_{t}$. However, as job processing concludes, $a_{t}$ progressively diminishes until it reaches zero upon the completion of all jobs. Additionally, it is noteworthy that when selecting an action in the state $s_{t}$, the PFSP state transitions from $s_{t}$ to $s_{t+1}$, consequently generating a novel disjunctive graph.

State Transition: owing to the constant change in the PFSP environment, the scheduling environment advances from the state $s_{t}$ to the next decision step $s_{t+1}$, wherein the job under consideration transitions from $J_{i}$ to $J_{i+1}$. Designating the temporal inception of the initial state $s_{0}$ as $t_{0}=0$, the initiation of a state transition transpires upon the completion of the initial machine task. In the event that the time of transition is t in the state $s_{t}$, the reward acquired upon the environment’s transition to state $s_{t+1}$ subsequent to the agent’s execution of action $a_{t+1}$ is denoted as $r_{t+1}$.

Reward: the reward function is used to evaluate the agent’s behavior and guide the agent to choose the appropriate behavior for different states and optimize the policy. This article’s objective resides in the minimization of the PFSP makespan. According to the characteristics of the problem constraint and scheduling model, the earlier the processing time of each job on the first machine, the more compact the arrangement of the jobs and the shorter the completion time. However, the reward function that only gives feedback at the end of each round of scheduling makes it difficult for agents to understand how each action affects the global results. To surmount this challenge, we have devised a reward function that mitigates such limitations, as shown in Equation (1). The function first calculates the difference between the partial solutions between two consecutive steps t and t+1.

(1) $$r\left(a_{t},s_{t}\right)=I\left(s_{t}\right)-I\left(s_{t+1}\right)$$

$I\left(s_{t}\right)$ characterizes the solution quality in terms of the makespan. We define it as $I(s_{t})={max}_{ij}\{C_{t}(O_{ij},s_{t})\}$, where $C_{t}(O_{ij},s_{t})$ is the completion time until the machine j finishes the job i at step t. Obviously, $I\left(s_{T}\right)$ corresponds to the makespan of the terminal state $s_{T}$, where $s_{T}$ is the state when the schedule is completed, and all disjunctive arcs have directions at this time. Through iterative calculations, the cumulative reward $R\left(a_{t},s_{t}\right)=I\left(s_{0}\right)-I\left(s_{T}\right)$. Since $I\left(s_{0}\right)$ remains constant, the maximization of cumulative reward is related to the minimization of the makespan.

Policy: Upon completion of model training, the policy network yields a probability distribution for candidate jobs at each decision point. The agent selects the job with the highest probability at each decision step and feeds it into the network to derive the probability distribution for the next candidate job.

### 3.2. Policy Network Based on GIN

In the pursuit of solving PFSP using the MDP framework established in the preceding section, we employ the state representation method of disjunctive graphs as the input for the policy network. These disjunctive graphs encompass both the node features of the states and the structural information of the graphs. To more effectively extract structural features, a policy founded on graph networks is necessary to extract state information. This study introduces the recently proposed GIN [45] as an encoder, presenting a policy network rooted in GIN. The encoder first encodes the original disjunctive graph into an implicit vector containing state information, upon which the policy network bases its decision-making process.

#### 3.2.1. Policy Network

Graph encoder: we employ an encoder based on GIN for encoding. When tackling PFSP, the disjunctive graph encapsulates pertinent information such as task precedence constraints and processing times for each job on various machines. The representation of the state within the disjunctive graph exhibits dynamic fluctuations. GIN, a variant of GNN, possesses robust isomorphism verification and inference capabilities, rendering it well-suited for dynamic graphs. Embedding these pertinent details through GIN facilitates efficient scheduling for PFSP. To enhance generalization capabilities and minimize the frequency of policy network training, we deploy a GIN to encode and express $s_{t}$. GIN extracts the feature embedding of each node in a disjunctive graph in an iterative and nonlinear way. For a disjunctive graph G=(O,C,D) representing the real scheduling state, at each time step *t*, each node o∈O undergoes encoding via *L* layers of GIN, denoted as $h_{o,t}^{\left(l\right)}$, as defined in Equation (2). Here, ${MLP}_{\theta_{l}}^{\left(l\right)}$ corresponds to the l-th layer of a multi-layer perceptron (MLP), $\theta_{l}$ represents the layer’s parameters, ${MLP}_{\theta_{l}}^{\left(l\right)}$ is used to iterate l and normalize the batch; $\varphi^{\left(l\right)}$ denotes the learning parameters, which is an arbitrary parameter that can be learned, and δ(o) signifies the neighborhood set of o.

(2) $$h_{o,t}^{\left(l\right)}={MLP}_{\theta_{l}}^{\left(l\right)}((1+\varphi^{\left(l\right)})\times h_{o,t}^{\left(l-1\right)}+\sum_{p\in \delta (o)}h_{o,t}^{\left(l-1\right)})$$

After undergoing l iterations of update, obtain the global representation of the entire disjunctive graph using the mean pooling function, as illustrated by Equation (3). After coding by the encoder, we can parameterize the policy π*(*$a_{t}|s_{t}$*)* into a graph neural network $\pi_{\theta }$*(*$a_{t}|s_{t}$*)* with trainable parameters θ, which makes our model able to deal with unknown scale instances.

(3) $$h_{G}=\frac{1}{|O|\sum_{o\in O}h_{o,t}^{\left(l-1\right)}}$$

Action selection network: the encoder typically comprises neural networks. Considering model complexity, we employ an MLP as the action selection network. After encoding, the disjunctive graph yields a representation $h_{G}$ of the state. Subsequently, the action selection network maps the state representation $h_{G}$ to a probability distribution over actions, employing the softmax function for output. In each decision step, the agent sequentially selects the optimal action based on the order of probabilities.

#### 3.2.2. Training Framework

Next, we elucidate the training framework for our algorithm. Due to the high variance exhibited by randomly generated training data, the training process becomes notably unstable. Furthermore, given the sensitivity of DRL to hyperparameter tuning, we employ the PPO algorithm [46] to mitigate the aforementioned challenges and train our model. Benefiting from the actor–critic (AC) architecture, PPO adeptly addresses continuous control problems. It stands as a typical same-track strategy algorithm, signifying that the policy improved in each round aligns with the policy utilized for sampling.

PPO is rooted in the AC framework, encompassing two networks: the actor, denoted by the policy network $\pi_{\theta }$($a_{t}|s_{t}$) than described above, and the critic, a value function network. Notably, both networks share a GIN. The agent selects actions from the policy network’s output, while the value function network evaluates actions. The actor component utilizes a policy function $\pi_{\theta }(s_{t}|a_{t})$ to delineate the relationship between states and actions. Meanwhile, the critic component employs the parameter ω within the action-state value function $Q_{\pi_{\theta }}(s_{t},a_{t})$ to guide the direction of policy updates, thereby crafting the $Q_{\pi_{\theta }}(s_{t},a_{t})$ function to appraise the execution of action $a_{t}$ given input state features $s_{t}$. $Q_{\pi_{\theta }}(s_{t},a_{t})$ is mainly used to calculate the dominance function together with the state value function. The equation of $Q_{\pi_{\theta }}(s_{t},a_{t})$ is Equation (4).

(4) $$Q_{\pi_{\theta }}(s_{t},a_{t})=E[U_{t}|S_{t}=s_{t},A_{t}=a_{t}]$$

where $Q_{\pi_{\theta }}(s_{t},a_{t})$ represents the long-term expected discount reward received after executing the action $a_{t}$ at state $s_{t}$. E(.) represents the expected value and $U_{t}$ represents the future return value from step t. The calculation equation of $U_{t}$ is Equation (5).

(5) $$U_{t}=r_{t}+\gamma r_{t+1}+{\gamma^{2}r}_{t+2}+\cdots$$

where γ is the discount factor ∈(0,1).

The two networks undergo parameter updates via alternating gradient descent. A comprehensive account of the PPO algorithm’s intricate training process is provided in Algorithm 1.
**Algorithm 1** PPO-Based Training Algorithm**Input:** discounting factor γ; clipping ratio ε; update epoch L; number of training steps E; critic network $v_{\emptyset }$; actor-network $\pi_{\theta }$, behavior actor-network $\pi_{\theta_{old}}$, where ${\theta =\theta }_{old}$; entropy loss coefficient $f_{e}$; value function loss coefficient $f_{v}$; policy loss coefficient $f_{p}$. 1    Initialize $\pi_{\theta }$, $\pi_{\theta_{old}}$, and $v_{\emptyset }$; 2    **for *e* = 1 to E** 3        Pick *N* independent scheduling instances from distribution *D*; 4        **for *n* = 1 to N** 5              **for t = 1 to …** 6                  sample $a_{n,t}$ based on $\pi_{\theta_{old}}\left(a_{n,t}|S_{n,t}\right)$; 7                  Receive reward $r_{n,t}$ and next state $S_{n,t+1}$; 8                  Compute the advantage function ${\hat{A}}_{n,t}$ and probability ratio $r_{n,t}(\theta )$. 9                  ${\hat{A}}_{n,t}$ = $\sum_{0}^{t}Y^{t}r_{n,t}-V_{\emptyset }(S_{n},t)$; 10                    $r_{n,t}(\theta )$=$\frac{\pi_{\theta }\left(a_{n,t}|S_{n,t}\right)}{\pi_{\theta_{old}\left(a_{n,t}|S_{n,t}\right)}}$ 11                    while $s_{n,t}$ is terminal do 12                          break; 13                  **end while** 14            **end for** 15            Compute the policy loss $L_{n}^{PPO}\left(\theta \right)$, the value function loss $L_{n}^{critic}\left(\emptyset \right)$ and the entropy loss $L_{n}^{S}\left(\theta \right)$. 16            $L_{n}^{PPO}\left(\theta \right)=\sum_{0}^{t}min(r_{n,t}\left(\theta \right){\hat{A}}_{n,t},clip(r_{n,t}\left(\theta \right),1-\varepsilon ,1+\varepsilon ){\hat{A}}_{n,t})$; 17            $L_{n}^{critic}\left(\emptyset \right)=\sum_{0}^{t}{\left(v_{\emptyset }\left(s_{n,t}\right)-{\hat{A}}_{n,t}\right)}^{2}$; 18            $L_{n}^{S}\left(\theta \right)=\sum_{0}^{t}S(\pi_{\theta }(a_{n,t}|s_{n,t}))$, where S(·) is entropy; 19            Total Losses: $L_{n}\left(\theta ,\emptyset \right)=f_{p}L_{n}^{PPO}\left(\theta \right)-{f_{v}L}_{n}^{critic}\left(\emptyset \right)+{f_{e}L}_{n}^{S}\left(\theta \right)$; 20        **end for** 21        for *l* = 1 to L 22            Update θ, ∅ with cumulative loss by Adam optimizer: 23            θ,$\;\emptyset \leftarrow Adam(\sum_{n=1}^{N}L_{n}\left(\theta ,\emptyset \right))$ 24        **end for** 25    $\theta_{old}\leftarrow \theta$ 26    **end for** 27    **Output:** Trained parameter set of θ.

**Figure 3:**
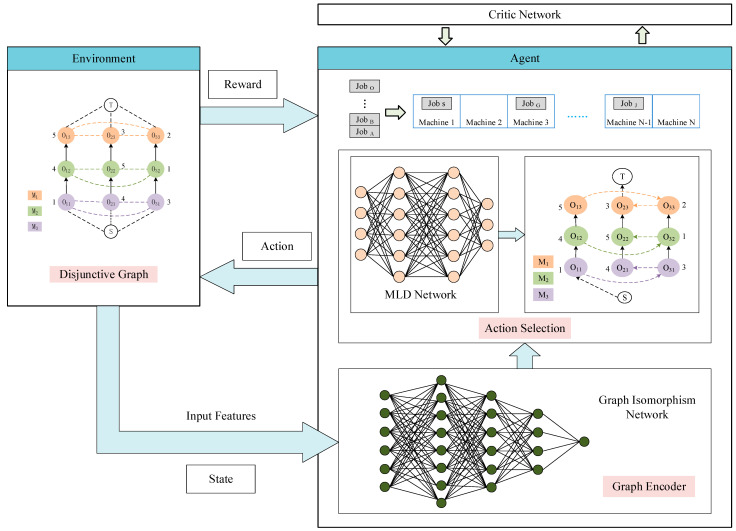
The DRL framework based on PPO.

Figure 3 illustrates the training framework of our model, showcasing the process by which the DRL approach proposed in this study addresses the scheduling challenges of PFSP. This reinforcement learning paradigm consists of an agent responsible for determining the order of job inputs and an environment, which captures the current state of PFSP using disjunctive graphs. Initially, the environment feeds the disjunctive graph, encompassing the machining status of various machines, into the graph encoder. This encoder transforms the original disjunctive graph into an implicit vector carrying state information. Subsequently, an action selection network based on an MLP generates a probability distribution over potential actions. The agent’s decision-making process involves selecting the optimal action based on the probabilities. This determines the job to be inputted into the current pipeline state. The environment confers rewards to the agent based on the decisions made, iterating through this process until scheduling for all pending jobs is completed.

## 4. Numerical Experiment

These data samples employed for model training are randomly generated, with a total count of 10,000. Among these, the number of machines is set at five. The total job count for each individual sample adheres to a uniform distribution within the range of [5, 100], while the processing times follow a uniform distribution within the interval [0, 100]. To ascertain the efficacy of our proposed methodology, we conducted tests on both randomly generated instances and the Taillard benchmark dataset [47]. Comparative analyses were performed against heuristic algorithms SPT and NEH, along with metaheuristic algorithms Ant Colony Optimization (ACO) and Genetic Algorithms (GA). Additionally, we contrasted the experimental outcomes against DRL algorithms Dueling Double Deep Q Network (D3QN) and PPO, which do not employ disjunctive graph-based state representations. Among them, D3QN is a variant of the Dueling DQN algorithm, which incorporates the idea of the Double DQN algorithm on the basis of the Dueling DQN algorithm.

### 4.1. Experimental and Parameter Settings

We conducted an extensive series of experiments using the methodology we proposed to solve the PFSP in order to validate its efficiency and effectiveness. All experiments were implemented in Python 3.8, running on a computer equipped with an AMD Ryzen 7 5800 H CPU clocked at 3.20 GHz and an NVIDIA RTX 3050 Ti GPU. Appropriate parameter configurations are crucial for the successful training of the model. Each MLP within the GIN architecture comprises two hidden layers, with each layer having a dimension of 64. Similarly, the MLP within the action selection network contains two hidden layers, each with a dimension of 32. The remaining hyperparameters during the training process are detailed in Table 1.

### 4.2. Performance Metrics

For the optimization problem of the PFSP studied in this article, we employ two metrics to assess the quality of both baseline methods and our proposed approach. These metrics encompass makespan and computational time. Makespan signifies the maximum completion time expended for resolving this scheduling quandary. In addition to comparing the magnitudes of makespan across various algorithms, we also employ the Relative Percentage Deviation (RPD) [48] to gauge the algorithms’ performance in terms of makespan, as denoted by Equation (6). Herein, $C_{max}^{best}$ represents the currently best makespan for this problem, while $C_{max}$ corresponds to the makespan computed by the current algorithm for this issue. It is worth noting that algorithms with lower RPD values exhibit superior performance compared with those with higher RPD values. In the practical realm of factory production, the time taken to obtain solutions for problems also assumes importance. Swift discovery of resolutions to production issues accelerates the process of restoring production to full capacity. This, in turn, aids in more effectively meeting production objectives and customer demands. Hence, we also consider the model’s computational time as one of the metrics for evaluating model performance.

(6) $$RPD=\frac{C_{max}-C_{max}^{best}}{C_{max}^{best}}\times 100$$

### 4.3. Computational Results of Randomly Generated Instances

We commence by subjecting our model to testing on randomly generated instances, ranging in size from 6 × 6 to 100 × 20. Eight examples with different scales are tested, and the processing times of the jobs on a single machine adhere to a uniform distribution within the interval [0, 100]. We tested different algorithms on these same randomly generated data at the same scale and compared the performance of each algorithm. We compare the test outcomes against those of SPT, NEH, ACO, GA, D3QN, and PPO algorithms, the latter of which does not employ a disjunctive graph representation of states. The makespan yielded by each algorithm is presented in Table 2, measured in hours (h). The bold typeface highlights the optimal results for each instance. As Table 2 elucidates, our model attains a lower makespan compared to all baseline methods. For instance, when faced with a problem of dimensions 100 × 20, our proposed model reduces makespan by 183.2 h, 59.7 h, 69.2 h, 58.3 h, 39.8 h, and 28.4 h, respectively, compared the six baseline methods. In a broader perspective, DRL algorithms D3QN and PPO outperform heuristic and metaheuristic approaches. The heuristic algorithm NEH holds a competitive stance against metaheuristic algorithms such as ACO and GA, while the performance of SPT, another heuristic algorithm, performs poorly.

Furthermore, it is noteworthy that as the scale of the problem expands, the disparity between the baseline methods and the model presented in this study intensifies. For instance, in the case of the D3QN algorithm, the makespan gap grows from 1.9 h to 39.8 h. This underscores the superior generalization prowess of our model when confronted with larger-scale instances in comparison to the baseline approaches.

In order to encompass the disparities in makespan achieved by the model from multiple perspectives, we employ the RPD in Table 3 to gauge the performance of each algorithm in terms of makespan across various instance scales. Given that the approach proposed in this study yields a makespan smaller than that of the baseline methods for every test instance, the RPD of our model is uniformly zero across all instances, signifying that it consistently outperforms the baseline methods in this regard. For instance, in the case of an instance size of 100 × 5, the disparities in RPD between the six baseline methods and our model are as follows: 2.8238, 1.0818, 1.0501, 1.0613, 0.7293, and 0.4476, respectively. Overall, the RPD of DRL algorithms is comparatively lower than that of heuristic and metaheuristic algorithms. Among DRL algorithms, the PPO algorithm, which does not employ disjunctive graph representations for states, exhibits a marginal advantage over the D3QN algorithm. Metaheuristic algorithms, on the whole, surpass heuristic algorithms, with NEH closely approaching the RPD values of metaheuristic algorithms.

The performance of the scheduling model is not solely contingent upon the quality of solution generation; the swiftness of solution generation also stands as a significant metric. Table 4 presents the computational times for both baseline methods and the method proposed in this study, and the unit of data in the table is seconds (s). It is evident from the table that SPT achieves nearly instantaneous resolution for all problems. Apart from the SPT algorithm, the D3QN algorithm exhibits the fastest computational speed for problem sizes ranging from 10 × 10 to 50 × 5, surpassing our model in this regard. However, as the instance scales continue to escalate, our model demonstrates faster computational speed, further accentuated by the growing discrepancy in computational times between the baseline methods and our proposed approach. For instance, in the case of the PPO algorithm, which does not incorporate disjunctive graphs, the disparity in computational times increases from 0.04 s to 5.24 s. Benefiting from the characteristics of DRL algorithms, which can handle instances of all scales after a single training session, the computational time of DRL algorithms is superior to that of heuristic and metaheuristic algorithms. However, heuristic scheduling algorithms outpace metaheuristic algorithms in terms of computational efficiency.

### 4.4. Computational Results of Benchmark Instances

In this section, we juxtapose our model against six baseline methods, SPT, NEH, ACO, GA, D3QN, and PPO, without disjunctive graph state representation on the esteemed Taillard benchmark. We conduct comparative experiments on ten distinct instances from the Taillard benchmark, spanning dimensions of 20 × 5 to 200 × 10. To ensure the reliability of experimental outcomes, we employ the same trained model across these trials. Table 5 presents the experimental findings for our model and the six baseline methods.

While NEH, ACO, GA, D3QN, PPO, and our model exhibit identical results for an instance of size 20 × 5, the disparity between the baseline methods and our model becomes more pronounced as instance dimensions expand. For instance, in the case of the PPO algorithm without disjunctive graph usage, the discrepancy between it and our proposed model escalates from 0 to 33.3 h as the instance scale increases. When compared to heuristic and metaheuristic algorithms, DRL algorithms still demonstrate competitive performance, adept at adaptive problem-solving across varying scheduling contexts. Metaheuristic algorithms, on the whole, outperform heuristic algorithms, with NEH showcasing performance akin to metaheuristic counterparts.

Table 6 presents the RPD values of our model and various baseline algorithms on different scale instances from the esteemed Taillard benchmark, with the optimal results highlighted in bold. For an instance size of 20 × 5, all algorithms except SPT exhibit an RPD value of zero, signifying that, in this instance, all algorithms other than SPT achieve outcomes identical to the benchmark’s optimal results. As the instances grow larger, our model attains smaller RPD values compared to the baseline methods. For instance, when faced with an instance size of 200 × 10, the disparities in RPD between the six baseline methods and our model are as follows: 1.7563, 0.4456, 0.5631, 0.4904, 0.3803, and 0.3104, respectively. Across instances of the same scale, DRL algorithms consistently manifest lower RPD values compared to the other two categories of algorithms. Among DRL algorithms, the RPD value of the PPO algorithm without disjunctive graphs slightly surpasses that of D3QN. In contrast, the NEH RPD value closely approximates those of the metaheuristic algorithms ACO and GA, with SPT exhibiting the least favorable performance.

Similarly, we have also conducted a comparison of the computational times for each algorithm on the Taillard benchmark, as depicted in Table 7. The SPT algorithm continues to showcase near-instantaneous computational prowess. Apart from SPT, D3QN exhibits swifter computational abilities for smaller instances ranging from 20 × 5 to 50 × 5, as compared to the model proposed in this paper. However, as instance dimensions escalate from 50 × 10 to 200 × 10, our model demonstrates heightened computational speed. Furthermore, with an increase in instance size, the gap in computational time between D3QN and our model expands from 0.2 s to 4.96 s. DRL algorithms persist in manifesting quicker computational speeds than heuristic and metaheuristic methods, whereas the computational time of metaheuristic algorithms is longer than that of heuristic algorithms.

### 4.5. Discussion

The SPT algorithm demonstrates instantaneous computational prowess both on randomly generated instances and the Taillard benchmark yet falls short in generating solutions of satisfactory quality. In contrast, the approach proposed in this study, while slightly slower in computation compared to SPT, proves quicker than other baseline methods. This trade-off remains acceptable in practical production contexts, further amplified by the superior solution quality generated by our proposed method in comparison to all baseline approaches. Furthermore, as the problem-solving dimensions expand, the disparity in makespan and computational time between the baseline methods and our proposed model also grows, underscoring the heightened robustness of our method over baseline approaches in both test scenarios. These experiments verify the smoothness of our model in solving PFSF problems and further prove that the correlation between the MDP model and PFSF is effective. Consequently, a comprehensive evaluation indicates the superiority of our model over all heuristic scheduling rules, metaheuristic algorithms, and DRL algorithms.

Benefiting from the inherent self-learning capacity of DRL algorithms and their ability to handle instances of varying scales following a single training session, these algorithms outshine heuristic and metaheuristic methods. Notably, the model proposed in this paper exhibits superior performance in both makespan and computational time compared to the PPO algorithm that abstains from disjunctive graph state representation. Our model’s prowess in addressing PFSP can be attributed to two factors: First, the disjunctive graph-based state representation method provides a more comprehensive depiction of scheduling states, proving efficacious even when dealing with intricate and sizable PFSP instances. Second, the strategy network founded on GIN more effectively absorbs the underlying information of graph structures. Additionally, the encoding process within the graph encoder primarily relies on matrix parallel computation, enhancing the computational efficiency of the model.

## 5. Conclusions and Future Work

In this study, we propose a novel end-to-end DRL framework to address the PFSP. Our initial step involves constructing an MDP model for PFSP, meticulously elaborating on the definitions of states, actions, and rewards. Notably, we innovatively portray the PFSP environment using disjunctive graphs. To capture the underlying topological structure of disjunctive graphs, we engineer a strategy network rooted in GIN. This network adeptly extracts rich representational information from node embeddings. Training of this network is executed via the PPO algorithm. In assessing the performance of the proposed model, we employ makespan and computational time as evaluative benchmarks. Experimental validation takes place on both randomly generated instances and the Taillard public benchmark. Outcomes affirm our model’s superiority over heuristic, metaheuristic, and DRL-based baseline approaches. Moreover, the model’s seamless extensibility to larger problem instances without necessitating retraining underscores its commendable scalability. Given the rapid expansion of the manufacturing industry, characterized by heightened product complexity and demand, optimizing production line efficiency through advanced methods assumes paramount significance. Our model, empowered by its robust generalization capacity, can effectively confront this challenge, making it a formidable tool for enhancing production efficiency amid evolving industrial landscapes.

Although we have validated the efficacy of the method proposed in this study in outperforming baseline approaches, there remains some disparity between the obtained results and the standard outcomes for each instance in the Taillard benchmark test. Moving forward, we shall continue enhancing our model and subject it to testing on more and larger benchmark data sets. To strike a balance between computation time and result quality, we have employed a simple Multi-layer Perceptron (MLP) as the action selection network. However, the inclusion of more intricate modules within the action selection network will undoubtedly contribute to the amplification of the model’s performance. In the future, we will further delve into the exploration of the action selection network, aiming to advance result quality while maintaining an acceptable level of computational efficiency. Additionally, this study primarily delves into the realm of single-objective optimization, with the objective of minimizing makespan. Yet, practical production scenarios often entail the consideration of multiple objectives, such as the energy consumption of equipment. In our subsequent endeavors, we will conduct a more profound analysis of the PFSP and undertake refinements to our model, thereby addressing the broader spectrum of multi-objective optimization challenges prevalent in real-world production settings.

## Figures and Tables

**Table 1:** Hyperparameter configuration.

Hyperparameter	Value
Learning rate	$10^{-4}$
Learning rate decay factor	0.98
Learning rate decay step	3000
The clipping parameter	0.2
The policy loss coefficient	2
Optimizer	Adam
Batch size	128

**Table 2:** The makespan of each algorithm on randomly generated instances (h).

Size	Heuristic		Metaheuristic		Reinforcement Learning		Ours
	SPT	NEH	ACO	GA	D3QN	PPO	
10 × 10	1086.5	1085.4	1086.2	1085.5	1085.5	1085.2	**1083.6**
15 × 15	1692	1667.2	1665.8	1667.2	1657.3	1655	**1646.3**
20 × 20	2301.9	2243.9	2247	2251.6	2217.4	2215.1	**2201.7**
50 × 5	2921.8	2865.1	2862.5	2867.2	2834.5	2831.7	**2816.4**
50 × 10	3254.5	3193.7	3192.2	3195.1	3169.2	3162.5	**3144.2**
50 × 20	3988.2	3891.5	3889.3	3894	3875.4	3861.7	**3839.4**
100 × 5	5513	5419.6	5417.9	5418.5	5400.7	5385.6	**5361.6**
100 × 20	6772.3	6648.8	6658.3	6647.4	6628.9	6617.5	**6589.1**

**Table 3:** The RPD of each algorithm on randomly generated instances.

Size	Heuristic		Metaheuristic		Reinforcement Learning		Ours
	SPT	NEH	ACO	GA	D3QN	PPO	
10 × 10	0.2676	0.1661	0.2399	0.1753	0.1753	0.1477	**0**
15 × 15	2.7759	1.2695	1.1845	1.2695	0.6682	0.5285	**0**
20 × 20	4.5510	1.9167	2.0575	2.2664	0.7131	0.6086	**0**
50 × 5	3.7424	1.7292	1.6368	1.8037	0.6427	0.5432	**0**
50 × 10	3.5080	1.5743	1.5266	1.6189	0.7951	0.5820	**0**
50 × 20	3.8756	1.3570	1.2997	1.4221	0.9376	0.5808	**0**
100 × 5	2.8238	1.0818	1.0501	1.0613	0.7293	0.4476	**0**
100 × 20	2.7803	0.9060	1.0502	0.8848	0.6040	0.4310	**0**

**Table 4:** The computation time of each algorithm on randomly generated instances (s).

Size	Heuristic		Metaheuristic		Reinforcement Learning		Ours
	SPT	NEH	ACO	GA	D3QN	PPO	
10 × 10	**0**	1.86	5.49	3.84	0.77	0.81	0.77
15 × 15	**0**	2.37	6.02	4.27	1.19	1.44	1.28
20 × 20	**0**	2.59	8.37	5.88	1.35	1.64	1.44
50 × 5	**0**	2.61	10.15	6.35	1.41	1.68	1.46
50 × 10	**0**	4.85	13.64	7.75	2.75	2.99	2.39
50 × 20	**0**	6.97	18.71	9.11	4.49	5.33	3.42
100 × 5	**0**	7.84	19.21	9.68	5.51	6.25	3.79
100 × 20	**0**	13.05	25.17	16.27	10.53	11.71	6.47

**Table 5:** The makespan of each algorithm on Taillard benchmark (h).

Problem Instance	Size	Heuristic		Metaheuristic		Reinforcement Learning		Ours
		SPT	NEH	ACO	GA	D3QN	PPO	
Ta010	20 × 5	1149.4	**1108**	**1108**	**1108**	**1108**	**1108**	**1108**
Ta020	20 × 10	1695.3	1665.9	1662.5	1661.2	1658.7	1646.5	**1639.8**
Ta030	20 × 20	2313.7	2270.3	2269.2	2265.4	2263.6	2251	**2242.2**
Ta040	50 × 5	2957.1	2893.6	2887.5	2884.4	2882.5	2869.2	**2858.6**
Ta050	50 × 10	3261.3	3190.6	3182.1	3179.5	3181	3165.6	**3153.1**
Ta060	50 × 20	3996.5	3920.1	3917.6	3914.1	3908.7	3892.5	**3879.4**
Ta070	100 × 5	5531.8	5443.5	5441.6	5437	5435.3	5418.6	**5402**
Ta080	100 × 10	6093.2	5982.3	5982.1	5979.4	5972.6	5959.1	**5937.5**
Ta090	100 × 20	6785.4	6670.8	6679.2	6673.5	6661.2	6654.1	**6624.3**
Ta100	200 × 10	10,975.6	10,835	10,847.6	10,839.8	10,828	10,820.5	**10,787.2**

**Table 6:** The RPD of each algorithm on the Taillard benchmark.

Problem Instance	Size	Heuristic		Metaheuristic		Reinforcement Learning		Ours
		SPT	NEH	ACO	GA	D3QN	PPO	
Ta010	20 × 5	3.7365	**0**	**0**	**0**	**0**	**0**	**0**
Ta020	20 × 10	6.5556	4.7077	4.4940	4.4123	4.2552	3.4884	**3.0673**
Ta030	20 × 20	6.2305	4.2378	4.1873	4.0129	3.9302	3.3517	**2.9477**
Ta040	50 × 5	6.2940	4.0115	3.7922	3.6808	3.6125	3.1344	**2.7534**
Ta050	50 × 10	4.7303	2.4599	2.1869	2.1034	2.1516	1.6570	**1.2556**
Ta060	50 × 20	5.4207	3.4054	3.3395	3.2472	3.1047	2.6774	**2.3318**
Ta070	100 × 5	3.8251	2.1678	2.1321	2.0458	2.0139	1.7005	**1.3889**
Ta080	100 × 10	3.9795	2.0870	2.0836	2.0375	1.9215	1.6911	**1.3225**
Ta090	100 × 20	3.6889	1.9377	2.0660	1.9789	1.7910	1.6825	**1.2271**
Ta100	200 × 10	2.3175	1.0068	1.1243	1.0516	0.9415	0.8716	**0.5612**

**Table 7:** The computation time of each algorithm on Taillard benchmark (s).

Problem Instance	Size	Heuristic		Metaheuristic		Reinforcement Learning		Ours
		SPT	NEH	ACO	GA	D3QN	PPO	
Ta010	20 × 5	**0**	1.71	5.28	3.79	0.71	0.79	0.75
Ta020	20 × 10	**0**	2.15	5.85	4.31	1.14	1.36	1.24
Ta030	20 × 20	**0**	2.47	8.79	5.86	1.3	1.51	1.41
Ta040	50 × 5	**0**	2.59	9.36	6.29	1.37	1.59	1.43
Ta050	50 × 10	**0**	4.18	14.25	7.73	2.62	2.97	2.42
Ta060	50 × 20	**0**	6.74	17.53	9.02	4.61	5.36	3.45
Ta070	100 × 5	**0**	7.31	18.02	9.63	5.45	6.28	3.74
Ta080	100 × 10	**0**	10.86	21.03	12.94	7.64	9.3	4.49
Ta090	100 × 20	**0**	12.97	24.49	15.65	10.6	11.67	6.62
Ta100	200 × 10	**0**	24.79	36.31	30.13	23.11	25.05	18.15

## Data Availability

Not applicable.
